# Reaction of hydroxylated naphtoquinones/antraquinones with pentafluoropyridine

**DOI:** 10.1186/s40064-016-1708-5

**Published:** 2016-02-01

**Authors:** Khalil Beyki, Reza Haydari, Malek Taher Maghsoodlou

**Affiliations:** Department of Chemistry, Faculty of Science, University of Sistan and Baluchestan, P. O. Box 98135-674, Zahedan, Iran

**Keywords:** Pentafluoropyridine, Synthesis, Hydroxylated naphtoquinones, Hydroxylated antraquinones

## Abstract

In this study, the reaction of pentafluoropyridine with hydroxylated naphtoquinones and hydroxylated antraquinones was investigated under basic conditions in DMF. One or two hydroxyl group in naphtoquinones and antraquinones react with pentafluoropyridine to give mono and di-tetrafluoropyridyl naphtoquinones/antraquinones. All the compounds were characterized using ^1^H, ^13^C and ^19^F-NMR spectroscopy.

## Background

Recently, organofluorine compounds have been used as building blocks in the pharmaceutical industry and in material science due to their unique properties (Matthew et al. 2010). In pharmacology and medicinal researches, it is common to substitute hydrogen with fluorine atom for increasing the lipophilicity and the biological activity of drugs (Anatoliy et al. 2009). Various multi-functional pyridine derivatives and the construction of new heterocyclic drug systems could be accessed from the high electron efficiency of pentafluoropyridine and appropriate nucleophile in simple reaction conditions (Satoru et al. 2006; Cindy et al. 2010). The reactions of pentafluoropyridine with nucleophiles occurs in the most activated 4-position of pentafluoropyridine to give products arising from the substitution of fluorine, located para to ring nitrogen to give a range of 4-substituted tetrafluoropyridine systems (Mark et al. 2012). Previously, we reported the synthesis of 4-substituted 2,3,5,6-tetrafluoropyridine derivatives by the reaction of pentafluoropyridine with malononitrile, 1-tetrazole-5-thiol, and piperazine (Beyki et al. 2015). In this paper, we have recently reported the reaction of pentafluoropyridine with a very important class of biologically active compounds (Elias and Alexandros 2002), hydroxylated naphtoquinones and hydroxylated antraquinones. This allows the synthesis of a wide range of 4-substituted tetrafluoropyridine.

## Results and discussion

The reaction of pentafluoropyridine **1** with an equivalent of 5,8-dihydroxynaphthalene-1,4-dione **2** in the presence of potassium carbonate in DMF gave a mixture of two products, **2a** and **2b** arising from the displacement of the 4-fluor pyridine ring with naphtoquinones.

**Fig. 1:**
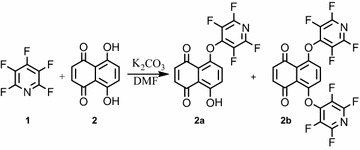
Reaction of pentafluoropyridine with 5,8-dihydroxynaphthalene-1,4-dione

In hydroxylated naphtoquinones **2**, the hydroxyl group deprotonates by potassium carbonate and attacks the para position of pentafluoropyridine to give **2a** (Fig. 1). In **2b**, both hydroxyl groups deprotonate and attack the para position of the pentafluoropyridine. The purification of **2a** and **2b** was achieved by column chromatography using ethyl acetate/n-hexane (1:10). The identification of **2a** was done by ^19^F NMR analysis, in which the resonance attributed to the fluorine located in ortho ring nitrogen has a chemical shift of −87.6 ppm and the fluorine located in meta to ring nitrogen occurs at −162.0 ppm. The two resonances by ^19^F NMR and their chemical shift indicate the displacement of fluorine atoms attached to the para position of pyridine ring. In ^1^H NMR of **2a**, the protons of the phenyl ring were observed at δ = 7.2–8.1 and hydroxyl group (OH) at 6.1 ppm. The mass spectrum of **2a** displayed the molecular ion peak (M^+^) at m/z = 339, which is consistent with the proposed structure.

In **2b**, ^19^F NMR spectroscopy shows two resonances at −83.5 and −84.3 ppm attributed to the fluorine located in the ortho position of the two ring nitrogen and the two resonances at −135.4 and −139.4 ppm attributed to the fluorine located in the meta position of two tetrafluoropyridine. In **2b**, the four resonances by ^19^F NMR indicate the two rings of pentafluoropyridine displacement of fluorine atoms at the para positions of pyridine rings. The ^1^H, ^13^C NMR confirmed the structure of **3b**. The protons of the phenyl ring were observed at 7.2 ppm. In MS spectrum, the molecular ion M^+^ +1 at m/z = 490 was observed.

**Fig. 2:**
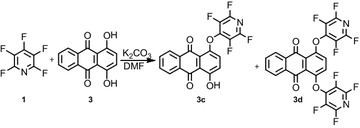
Reaction of pentafluoropyridine with 1,4-dihydroxyanthracene-9,10-dione

The reaction of pentafluoropyridine **1** with an equivalent of 1,4-dihydroxyanthracene-9,10-dione **3** in the presence of potassium carbonate as the base in DMF solvent gave **3c** and **3d** in 41 and 11 % yield respectively (Fig. 2). In **3c**, a hydroxyl group of **3** attacks the para position of the pyridine ring, and in **3d,** the two hydroxyl groups attack the most activated para position of pentafluoropyridine. The purification of **3c** and **3d** was achieved by column chromatography using ethyl acetate/n-hexane (1:10). The identification of **3c** was done from the ^19^F NMR analysis in which the resonance attributed to the fluorine atom located in the ortho positions had a chemical shift of −88.2 ppm. The corresponding resonance for the meta to ring nitrogen in **3c** occurs at −158.1 ppm. The ^1^H NMR spectra of compound **3c** showed an H broad signal at 4.2 ppm for OH group, and the protons of the phenyl ring were observed at δ = 7.2–8.3 ppm. In the MS spectrum of **3c,** the molecular ion M^+^ at m/z = 389 was observed.

Mass spectrometry and ^19^F NMR confirmed the product of **3d** as a di-tetrafluoropyridine system. The ^19^F NMR **3d** showed two resonances at −87.5 and −91.8 ppm attributed to the fluorine located in the ortho to ring nitrogen and the two resonances at −156.5 and −162.6 ppm attributed to the meta to ring nitrogen. Other spectroscopic techniques were consistent with the structures proposed. In ^1^H NMR, the spectra protons of the phenyl ring were observed at 7.5–8.7 ppm. The mass spectrum of **3d** displayed the molecular ion peak (M^+^) at m/z = 538, which is consistent with the proposed structure.

**Fig. 3:**
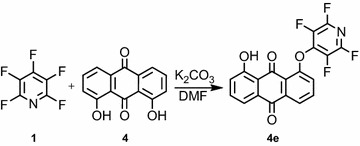
Reaction of pentafluoropyridine with 1,8-dihydroxyanthracene-9,10-dione

Also, we observed that the reaction of pentafluoropyridine **1** with 1,8-dihydroxyanthracene-9,10-dione **4** in the presence of potassium carbonate in DMF solvent gave **4e**. In basic conditions, the hydroxyl group of **4** deprotonation and attack on the para position of pentafluoropyridine gave **4e** (Fig. 3). The structure of compounds **4e** was confirmed by the NMR spectroscopic data and the MS analysis. In particular, the ^19^F NMR spectroscopy showed the chemical shift of fluorine atoms attached to the ortho and the meta position, observed respectively at −91.6 and −142.5 ppm. In ^1^H NMR, the protons of OH were observed at 4.2 ppm, and the protons of the phenyl ring were observed at 7.0–8.2 ppm. In the MS spectrum, the molecular ion M^+^ at m/z = 389 was observed.

## Conclusion

We showed that one or two hydroxyl groups in hydroxylated naphtoquinones and hydroxylated antraquinones can react with the pentafluoropyridineto afford of mono and di-2,3,5,6-tetrafluoropyridine naphtoquinones/antraquinones.

## Experimental

All materials and solvents were purchased from Merck and Aldrich and were used without any additional purification. The melting points of the products were determined in open capillary tubes using BAMSTEAB Electrothermal apparatus model 9002. Mass spectra were taken by a Micro mass Platform II: EI mode (70 eV). Silica plates (Merck) were used for TLC analysis.

### Preparation of 5-hydroxy-8-(perfluoropyridin-4-yloxy) naphthalene-1,4-dione 2a and 5,8-bis(perfluoropyridin-4-yloxy)naphthalene-1,4-dione 2b

Pentafluoropyridine **1** (0.16 g, 1 mmol), 5,8-dihydroxynaphthalene-1,4-dione **2** (0.19 g, 1 mmol) and potassium carbonate (0.11 g, 1.0 mmol) were stirred together in DMF (5 mL) at reflux temperature for 8 h. After completion of the reaction (indicated by TLC), reaction mixture was evaporated to dryness and water (10 mL) was added and extracted with dichloromethane (2 × 10 mL) and ethylacetate (2 × 10 mL). The mixture was filtered, volatiles evaporated and the residue purified by column chromatography on silica gel using ethyl acetate/n-hexane (1:10).

#### *5*-*hydroxy*-*8*-*(perfluoropyridin*-*4*-*yloxy)naphthalene*-*1,4*-*dione***2a**

(0.1 g, 29 %) as an orange solid; mp 240 °C dec; ^1^H NMR (CDCl_3_): δ (ppm) 4.1 (1H, s, OH), 7.2–8.3 (4H, m, Ar–H), ^19^F NMR (DMSO): δ (ppm) −87.6 (2F, s, F-2,6), −162.0 (2F, s, F-3,5), ^13^C NMR (CDCl_3_): δ (ppm) 115.3, 117.2, 119.7, 120.3, 125.4, 128.3, 131.0, 137.5, 150.3, 159.2, 169.2, 170.1, 171.8, MS (EI), m/z (%) = 341 (M^+^) (32 %) 318, 309, 301, 291, 272, 255, 246, 224, 198, 180, 152, 126, 106, 86, 58, 44.

#### *5,8*-*bis(perfluoropyridin*-*4*-*yloxy)naphthalene*-*1,4*-*dione***2b**

(0.08 g, 16 %) as an orange/yellow solid; mp 330 °C dec; ^1^H NMR (CDCl_3_): δ (ppm) 7.2 (5H, m, Ar–H), ^19^F NMR (CDCl_3_): δ (ppm) −83.5 (2F, s, F-2,6), −84.2 (2F, s, F-2^′^,6^′^), −135.4 (2F, s, F-3,5), −139.4 (2F, s, F-3^′^,5^′^). ^13^C NMR (CDCl_3_): δ (ppm) 91.8, 128.8, 130.9, 178.0, MS (EI), m/z (%) = 490 (M^+^ +2) (25 %), 461, 443, 409, 384, 369, 350, 331, 318, 302, 268, 255, 239, 212, 181, 167, 149, 136, 104, 90, 77, 57, 43.

### Preparation of 1-hydroxy-4-(perfluoropyridin-4-yloxy) anthracene-9,10-dione 3c and 1,4-bis(perfluoropyridin-4-yloxy)anthracene-9,10-dione 3d

Pentafluoropyridine **1** (0.16 g, 1 mmol), 1,4-dihydroxyanthracene-9,10-dione **3** (0.24 g, 1 mmol) and potassium carbonate (0.11 g, 1.0 mmol) were heated in DMF (5 mL) at reflux for 12 h (monitored by TLC). After completion of the reaction, the solvent was evaporated to dryness; water (10 mL) was added and extracted with dichloromethane (2 × 10 mL) and ethylacetate (2 × 10 mL). The mixture was filtered, volatiles evaporated and the residue purified by column chromatography on silica gel using ethyl acetate/n-hexane (1:10).

#### *1*-*hydroxy*-*4*-*(perfluoropyridin*-*4*-*yloxy)anthracene*-*9,10*-*dione***3c**

(0.16 g, 41 %) as a yellow solid; mp 355 °C dec; ^1^H NMR (CDCl_3_): δ (ppm) 4.1 (1H, s, OH), 7.2-8.3 (6H, m, Ar–H), ^19^F NMR (CDCl_3_): δ (ppm) −88.2 (2F, s, F-2,6), −158.1 (2F, s, F-3,5),^13^C NMR (CDCl_3_): δ (ppm) 126.5, 127.0, 133.6, 133.9, 138.8, 145.4, 148.5, 160.9, 188.9, MS (EI), m/z (%) = 389 (M), 371, 354, 340, 321, 307, 290, 263, 237, 212, 195, 181, 157, 143, 125, 112, 91, 77, 57, 43.

#### *1,4*-*bis(perfluoropyridin*-*4*-*yloxy)anthracene*-*9,10*-*dione***3d**

(0.06 g, 11 %) as a red/black solid; mp 315 °C dec; ^1^H NMR (CDCl_3_): δ (ppm) 4.2 (1H, s, OH), 7.2–8.7 (6H, m, Ar–H), ^19^F NMR (CDCl_3_): δ (ppm) −87.5 (2F, s, F-2,6), −91.8 (2F, s, F-2^′^,6^′^), −156.5 (2F, s, F-3,5), −162.6 (2F, s, F-3^′^,5^′^),^13^C NMR (CDCl_3_): δ (ppm) 120.9, 122.2, 123.4, 123.6, 124.5, 127.1, 128.5, 129.1, 130.1, 132.4, 136.5, 139.2, 140.4, 146.4, 147.4, 166.1, 170.2, MS (EI), m/z (%) = 538 (M), 496, 480, 439, 411, 398, 384, 362, 347, 331, 316, 302, 283, 270, 255, 239, 225, 209, 196, 181, 167, 154, 129, 105, 86, 57, 43.

### Preparation of 1-hydroxy-8-(perfluoropyridin-4-yloxy)anthracene-9,10-dione 4e

Pentafluoropyridine **1** (0.16 g, 1 mmol), 1,8-dihydroxyanthracene-9,10-dione **4** (0.24 g, 1 mmol) and potassium carbonate (0.11 g, 1.0 mmol) were heated in DMF (5 mL) at reflux for 12 h (monitored by TLC). After completion of the reaction, the solvent was evaporated to dryness; water (10 mL) was added and extracted with dichloromethane (2 × 10 mL) and ethylacetate (2 × 10 mL). The mixture was filtered, volatiles evaporated and the residue purified by column chromatography on silica gel using ethyl acetate/n-hexane (1:8).

#### *1*-*hydroxy*-*8*-*(perfluoropyridin*-*4*-*yloxy)anthracene*-*9,10*-*dione***4e**

(0.09 g, 23 %) as a red solid; mp 340 °C dec; ^1^H NMR (CDCl_3_): δ (ppm) 4.3 (1H, s, OH), 7.1–8.0 (6H, m, Ar–H), ^19^F NMR (CDCl_3_): δ (ppm) −91.6 (2F, s, F-2,6), −162.5 (2F, s, F-3,5),^13^C NMR (CDCl_3_): δ (ppm) 120.9, 122.2, 123.2, 123.6, 124.5, 127.1, 128.8, 130.2, 130.9, 131.2, 132.4, 136.5, 139.2, 140.4, 146.4, 147.9, 161.0, 164.0, 167.8. MS (EI), m/z (%) = 389 (M^+^), 387, 370, 364, 360, 342, 316, 299, 281, 255, 240, 223, 202, 184, 169, 155, 141, 127, 101, 79, 57, 43.
